# *Tff3*^−/−^ Knock-Out Mice with Altered Lipid Metabolism Exhibit a Lower Level of Inflammation following the Dietary Intake of Sodium Chloride for One Week

**DOI:** 10.3390/ijms24087315

**Published:** 2023-04-15

**Authors:** Nikolina Kolobarić, Martina Mihalj, Nataša Kozina, Anita Matić, Zrinka Mihaljević, Ivana Jukić, Ines Drenjančević

**Affiliations:** 1 Department of Physiology and Immunology, Faculty of Medicine Osijek, J. J. Strossmayer University of Osijek, J. Huttlera 4, 31000 Osijek, Croatia; nbdujmusic@mefos.hr (N.K.); mmihalj@mefos.hr (M.M.); nkozina@mefos.hr (N.K.); anitaa3006@gmail.com (A.M.); zmihaljevic@mefos.hr (Z.M.); ivana.jukic@mefos.hr (I.J.); 2 Scientific Center of Excellence for Personalized Health Care, J. J. Strossmayer University of Osijek, Trg Svetog Trojstva 3, 31000 Osijek, Croatia; 3 Department of Dermatology and Venereology, Osijek University Hospital, J. Huttlera 4, 31000 Osijek, Croatia

**Keywords:** inflammation, integrins, knock-out, sodium chloride, trefoil factor-3

## Abstract

A high salt intake causes hemodynamic changes and promotes immune response through cell activation and cytokine production, leading to pro-inflammatory conditions. Transgenic *Tff3*^−/−^ knock-out mice (TFF3ko) (*n* = 20) and wild-type mice (WT) (*n* = 20) were each divided into the (1) low-salt (LS) group and (2) high-salt (HS) group. Ten-week-old animals were fed with standard rodent chow (0.4% NaCl) (LS) or food containing 4% NaCl (HS) for one week (7 days). Inflammatory parameters from the sera were measured by Luminex assay. The integrin expression and rates of T cell subsets of interest from the peripheral blood leukocytes (PBLs) and mesenteric lymph nodes (MLNs) were measured using flow cytometry. There was a significant increase in high-sensitivity C reactive protein (hsCRP) only in the WT mice following the HS diet, while there were no significant changes in the serum levels of IFN-γ, TNF-α, IL-2, IL-4, or IL-6 as a response to treatment in either study groups. The rates of CD4^+^CD25^+^ T cells from MLNs decreased, while CD3^+^γδTCR^+^ from peripheral blood increased following the HS diet only in TFF3ko. γδTCR expressing T cell rates decreased in WT following the HS diet. The CD49d/VLA-4 expression decreased in the peripheral blood leukocytes in both groups following the HS diet. CD11a/LFA-1 expression significantly increased only in the peripheral blood Ly6C^−^CD11a^high^ monocytes in WT mice following salt loading. In conclusion, salt-loading in knock-out mice caused a lower level of inflammatory response compared with their control WT mice due to gene depletion.

## 1. Introduction

Even though it is necessary for the proper functioning of the organism, the intake of dietary salt (sodium chloride; NaCl) should be maintained at under 5 g/per day according to the World Health Organization (WHO) [1,2,3,4]. A high salt intake and its detrimental effects have been extensively explored for several decades, considering its contribution to inflammation [5,6,7], cardiovascular disease development [8,9], and other related complications [10,11]. Salt intake causes hemodynamic and immune response changes and induces cell activation and cytokine production, leading to pro-inflammatory reactivity [5,7,12,13] and pro-atherogenic conditions [14,15,16,17].

Cell adhesion molecules (CAMs), expressed in circulating leukocytes and endothelium [18], play an important role in early-phase atherosclerotic changes [19,20,21]. The levels of adhesion molecules, especially vascular cell adhesion molecule-1 (VCAM-1), may be valuable risk predictors in cardiovascular (CV) events in both patients and healthy populations. For example, upregulation of the VCAM-1 expression appears to be associated with atherosclerotic lesions and plaque formation [20,22,23,24,25]. CAMs are involved in mediation of the inflammatory cell recruitment following stimuli, such as stress or infection [20,26]. Upon T cell activation with two signals, (1) T Cell Receptor (TCR) + Major Histocompatibility Complex (MHC) on Antigen-Presenting Cells (APC) and (2) integrin Leukocyte function-associated antigen 1 (LFA-1) + ligand Intracellular Cell Adhesion Molecule 1 (ICAM-1) interaction [27,28,29], there is a release of pro-inflammatory cytokines – interleukin-12 (IL-12), interferon-gamma (IFN-γ). On the other hand, the blockage of the interaction of integrin with its corresponding ligands can lead to cytokine profile alteration towards anti-inflammatory IL-4 and IL-10 production [30]. Taken together, factors (extrinsic, such as the dietary salt intake, or intrinsic, such as cell membrane fatty acid composition) that can modify CAMs expression and activation, ultimately may direct the immune reaction towards being pro-atherogenic or anti-atherogenic.

Previously, it was demonstrated that a short-term high-NaCl diet altered the leukocyte expression of *β1* and *β2* integrins in both healthy humans and Sprague-Dawley (SD) rats, suggesting that the dietary intake of NaCl led to leukocyte activation, adhesion, and migration [5]. A variety of factors can impact the immune system’s ability to mount an effective response. Interestingly, *trefoil factor 3* gene (*Tff3*) knock-out mice with an altered lipid metabolism seem to evade the detrimental effects of salt-loading, which was described by Kozina et al. [31], as being a result of a “complex interaction of gene depletion and diet”. Mice with depleted *Tff3* gene utilize glucose from the bloodstream more effectively and have a modified arachidonic acid (AA) metabolism characterized by reduced monounsaturated fatty acids (MUFA), increased polyunsaturated fatty acids (PUFA), and a change in the n-3/n-6 PUFA ratio in the liver, as opposed to the control, namely the wild-type (WT) mice [32]. The TFF3 protein has been shown to alter the gut microbiota and also regulate the innate immune response at the mucosal surfaces, which represent critical barriers against the invasion of pathogens [33,34,35]. Furthermore, its deficiency changes the vascular function and response to different stimuli, thus affecting the innate immune response, particularly inflammation [36].

Thus, the main objective of the present study was to determine the effect of 7-day dietary salt intake on leukocyte integrin expression, rates of lymphocyte populations of interest (CD25^+^CD4^+^ and CD3^+^γδTCR^+^), and inflammatory status (CRP; cytokines) in *Tff3* knock-out mice and their respective controls, namely the WT mice.

## 2. Results

### 2.1. Influence of High-Salt Diet on Inflammatory Markers in Tff3 Knock-Out Mice and Their Wild-Type Control, C57BL/6N Mice

**Figure 1:**
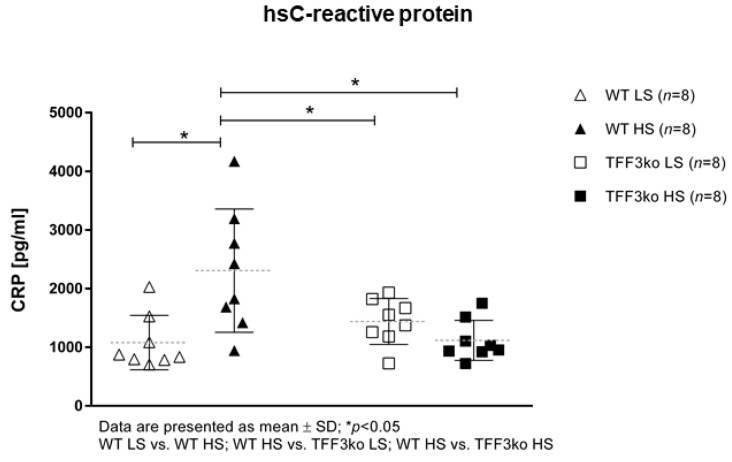
Serum concentrations of the inflammatory marker hsCRP in *Tff3*-deficient mice and their respective wild-type controls exposed to a 7-day high-salt diet. hsCRP—high-sensitivity C reactive protein; WT LS—wild-type (C57BL/6N) mice on a low-salt diet; WT LS—wild-type (C57BL/6N) mice on a high-salt diet; TFF3ko LS—*Tff3* knock-out mice on a low-salt diet; TFF3ko HS—*Tff3* knock-out mice on a high-salt diet. Data are presented as mean ± SD and were analyzed by one-way ANOVA or Kruskal–Wallis test where appropriate. * *p*-values less or equal 0.05 are considered significant.

**Figure 2:**
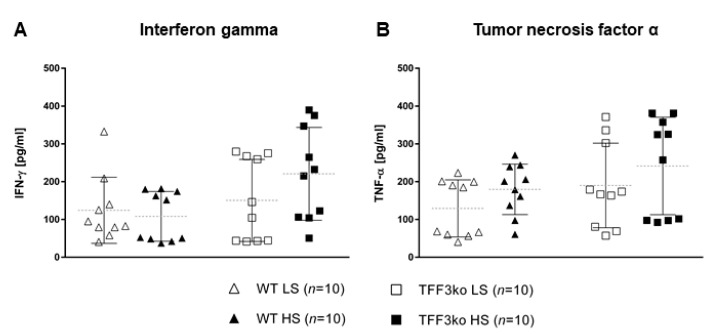
The serum concentrations of inflammatory markers IFN-γ (**A**) and TNF-α (**B**) in *Tff3*-deficient mice and their respective wild-type controls exposed to a 7-day high-salt diet. WT LS—wild-type (C57BL/6N) mice on a low-salt diet; WT LS—wild-type (C57BL/6N) mice on a high-salt diet; TFF3ko LS—*Tff3* knock-out mice on a low-salt diet; TFF3ko HS—*Tff3* knock-out mice on a high-salt diet; IFN-γ—interferon gamma; TNF-α—tumor necrosis factor alpha. Data are presented as mean ± SD and were analyzed by one-way ANOVA or Kruskal–Wallis test where appropriate.

**Figure 3:**
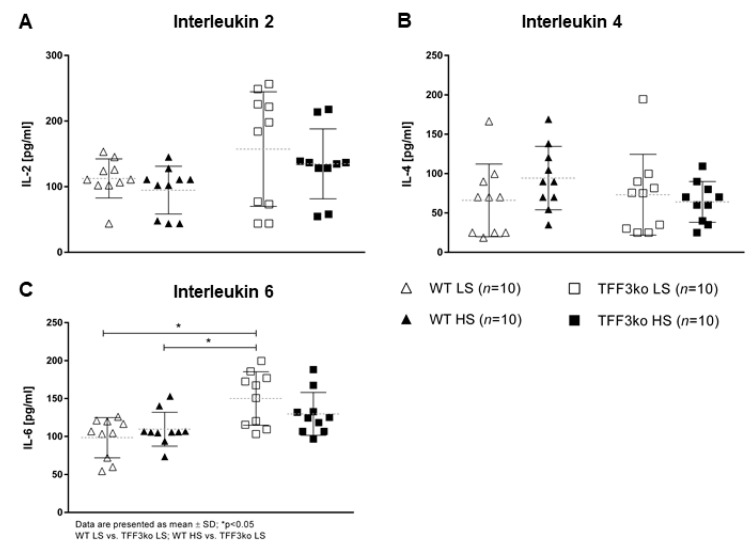
The serum concentrations of inflammatory markers IL-2 (**A**), IL-4 (**B**), and IL-6 (**C**) in *Tff3*-deficient mice and their respective wild-type controls exposed to a 7-day high-salt diet. IL—interleukin; WT LS—wild-type (C57BL/6N) mice on a low-salt diet; WT LS—wild-type (C57BL/6N) mice on a high-salt diet; TFF3ko LS—*Tff3* knock-out mice on a low-salt diet; TFF3ko HS—*Tff3* knock-out mice on a high-salt diet. Data are presented as mean ± SD and were analyzed by one-way ANOVA or Kruskal–Wallis test where appropriate. * *p*-values less or equal 0.05 are considered significant.

The level of high-sensitivity C-reactive protein (hsCRP) was significantly increased in the wild type (WT) high-salt (HS) group compared with WT low-salt (LS) (Figure 1). The serum IFN-γ and tumor necrosis factor-alpha (TNF-α) concentrations did not change significantly among groups after the protocol (Figure 2A,B). Meanwhile, the serum IL-6 concentrations were similar in the WT LS and WT HS groups, and between the *Tff3* knock-out (TFF3ko) LS and TFF3ko HS group, the IL-6 concentration was significantly increased in the TFF3ko LS group compared with the WT LS and WT HS groups (Figure 3C). No significant differences were found regarding IL-2 nor IL-4 serum concentrations after the protocol in either of the groups (Figure 3A,B).

### 2.2. Influence of High-Salt Diet on the Rates of CD25 and γδTCR-Expressing T Cells in Tff3 Knock-Out Mice and Their WT Control, C57BL/6N Mice

**Figure 4:**
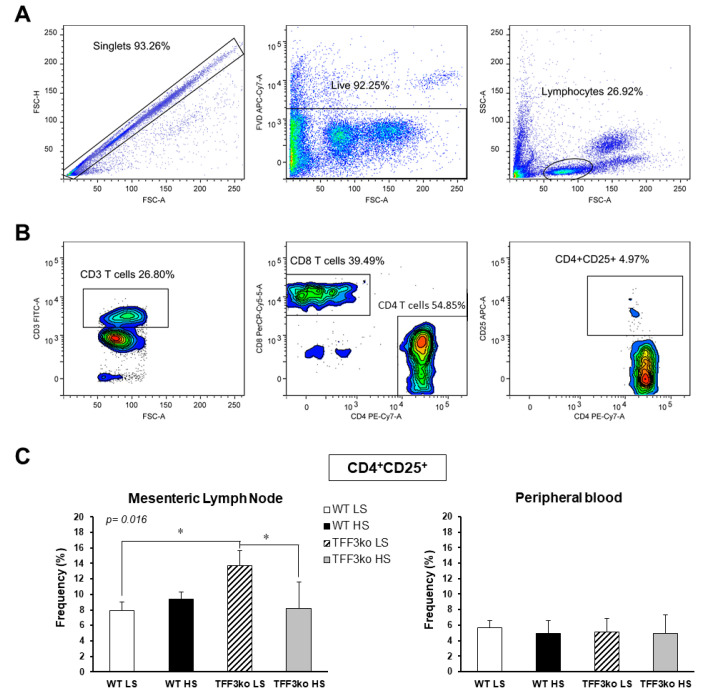
Rates of CD25 expressing CD4 T cells in the peripheral blood and mesenteric lymph nodes (MLNs) of *Tff3*-deficient mice and their respective wild-type controls exposed to a 7-day high-salt diet. Panel A and B demonstrate the representative gating strategy, including gating on live single cells and lymphocytes based on forward and side scatter (**A**), followed by gating on CD3^+^CD4^+^CD25^+^ T cells (**B**). (**C**) shows the changing rates of CD4^+^CD25^+^ in MLNs and peripheral blood in both study groups following the HS diet. MLNs—mesenteric lymph nodes; HS—high salt; WT LS—wild-type (C57BL/6N) mice on a low-salt diet; WT LS—wild-type (C57BL/6N) mice on a high-salt diet; TFF3ko LS—*Tff3* knock-out mice on a low-salt diet; TFF3ko HS—*Tff3* knock-out mice on a high-salt diet. Data are presented as mean ± SD and were analyzed by one-way ANOVA or Kruskal–Wallis test where appropriate. In some cases, the effect of the diet on a particular strain (TFFko or WT) was tested by Student’s *t*-test or Mann–Whitney U test; * *p*-values less or equal 0.05 are considered significant.

A representative gating strategy for CD4^+^CD25^+^ T cells in peripheral blood and mesenteric lymph nodes (MLNs) is shown in Figure 4A,B. TFF3ko LS mice had significantly higher rates of CD25^+^CD4^+^ T lymphocytes in mesenteric lymph nodes compared with their WT LS control. The HS diet significantly suppressed the CD25^+^CD4^+^ T lymphocyte rates in the TFF3ko HS compared with TFF3ko LS group in MLNs. No such effects were observed in the rates of CD4^+^CD25^+^ T cells in the peripheral blood (*p* = 0.016, Figure 4C).

**Figure 5:**
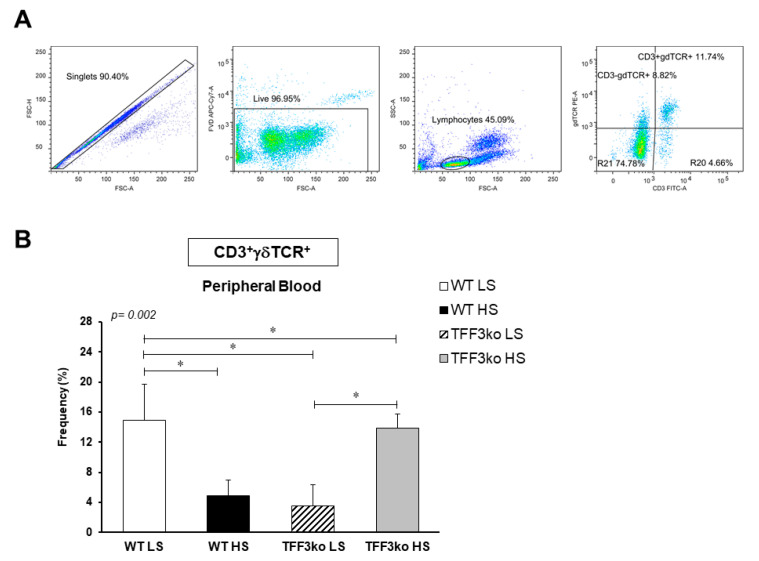
The effect of *Tff3* deficiency and a 7-day high-salt (HS) diet on the rates of CD3 T cells expressing gamma delta T-cell receptor in the peripheral blood. (**A**) demonstrates representative gating strategy, including gating on live single cells followed by gating on lymphocytes based on forward and side scatter and further analysis of their CD3 and γδTCR profiles. (**B**) shows the changing rates of CD3^+^γδTCR^+^ T cells in the study groups following the HS diet. HS—high salt; WT LS—wild-type (C57BL/6N) mice on a low-salt diet; WT LS—wild-type (C57BL/6N) mice on a high-salt diet; TFF3ko LS—*Tff3* knock-out mice on a low-salt diet; TFF3ko-HS—*Tff3* knock-out mice on a high-salt diet. Data are presented as mean ± SD and were analyzed by one-way ANOVA or Kruskal–Wallis test where appropriate. In some cases, the effect of the diet on a particular strain (TFFko or WT) was tested by Student’s *t*-test or Mann–Whitney U test; * *p*-values less or equal 0.05 are considered significant.

A representative gating strategy for CD3^+^γδTCR^+^ T cells is shown in Figure 5A. TFF3ko-LS had significantly lower rates of CD3^+^γδTCR^+^ cells compared with the WT-LS mice (Figure 5B). Furthermore, the 7-day HS diet reduced CD3^+^γδTCR^+^ frequency in WT mice while increasing it in the TFF3ko group (*p* = 0.002; Figure 5B).

### 2.3. Influence of a High-Salt Diet on LFA-1 and VLA-4 Expressions on Peripheral Blood Leukocytes and/or Mesenteric Lymph Nodes in Tff3 Knock-Out Mice and Their Wild-Type Control, C57BL/6N Mice

**Figure 6:**
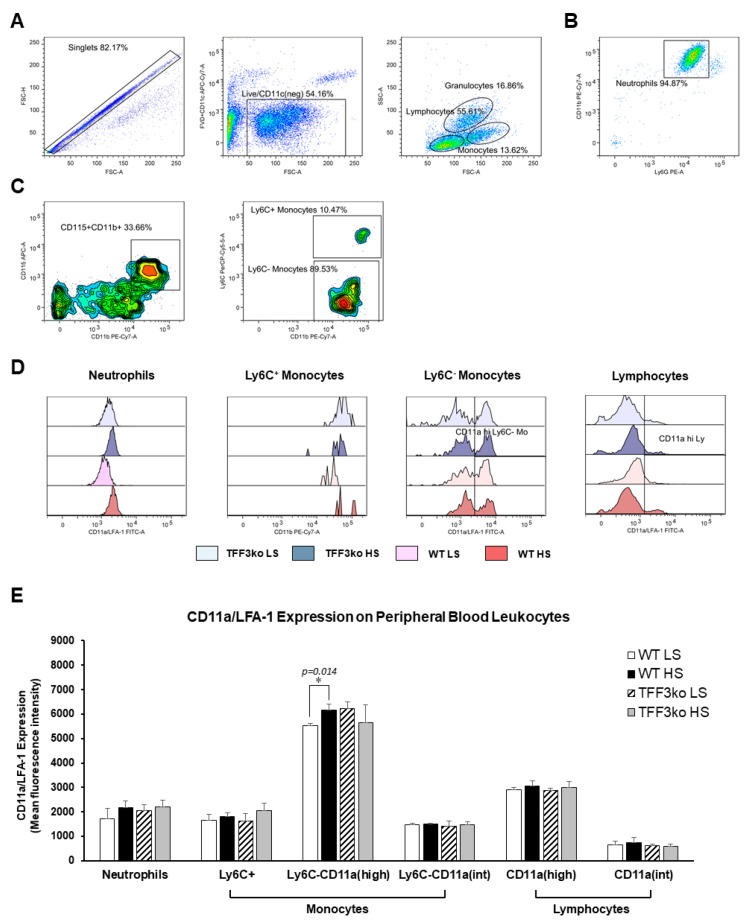
The effect of *Tff3* deficiency and a 7-day high-salt (HS) diet on the lymphocyte function-associated antigen 1 (LFA-1) expression in peripheral blood leukocytes (PBLs). (**A**–**C**) demonstrate the representative gating strategy, including gating on live single cells followed by gating on leukocyte subsets based on forward and side scatter. Neutrophils were further defined as Ly6C and CD11b double-positive cells among the granulocyte subpopulation (**B**), while the monocytes were defined as CD11b^+^CD115^+^ mononuclear cells that were additionally stratified based on the Ly6C expression (**C**). (**D**,**E**) show changing CD11a/LFA-1 expression rates on PBLs in the study groups following the HS diet. LFA-1—lymphocyte function-associated antigen 1; HS—high salt; PBLs—peripheral blood leukocytes; TFF3ko LS—*Tff3* knock-out mice on a low-salt diet; TFF3ko HS—*Tff3* knock-out mice on a high-salt diet; WT LS—wild-type (C57BL/6N) mice on a low-salt diet; WT HS—wild-type (C57BL/6N) mice on a high-salt diet. Data are presented as mean ± SD and were analyzed by one-way ANOVA or Kruskal–Wallis test where appropriate. In some cases, the effect of the diet on particular strain (TFFko or WT) was tested by Student’s *t*-test or Mann–Whitney U test. * *p*-values less or equal 0.05 are considered significant.

Figure 6 represents the gating strategy for CD11a/LFA-1 expression in the peripheral blood neutrophils, monocytes, and lymphocytes (panels A, B and C). There was no difference between the TFF3ko and WT mice in the expression of CD11a/LFA-1. In most leukocyte subsets, LFA-1 expression was increased after exposure to the HS diet; however, this was statistically significant only for the Ly6^−^ monocytes of the WT HS group, expressing high levels of LFA-1 (*p* = 0.014; Figure 6D,E).

**Figure 7:**
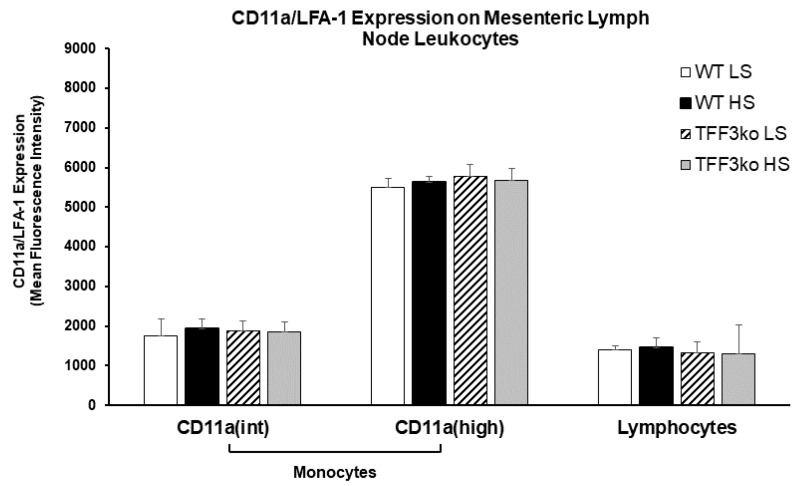
The effect of *Tff3* deficiency and a 7-day high-salt (HS) diet on the lymphocyte function-associated antigen 1 (LFA-1) expression in mononuclear cells isolated from mesenteric lymph nodes (MLNs). HS—high salt; LFA-1—lymphocyte function-associated antigen 1; MLNs—mesenteric lymph nodes; WT LS—wild-type (C57BL/6N) mice on a low-salt diet; WT LS—wild-type (C57BL/6N) mice on a high-salt diet; TFF3ko LS—*Tff3* knock-out mice on a low-salt diet; TFF3ko HS—*Tff3* knock-out mice ona high-salt diet. Data are presented as mean ± SD and were analyzed by one-way ANOVA or Kruskal–Wallis test where appropriate. In some cases, the effect of the diet on a particular strain (TFFko or WT) was tested by Student’s *t*-test or Mann–Whitney U test.

There were no significant differences regarding LFA-1 expression on MLNs among the studied groups (Figure 7).

**Figure 8:**
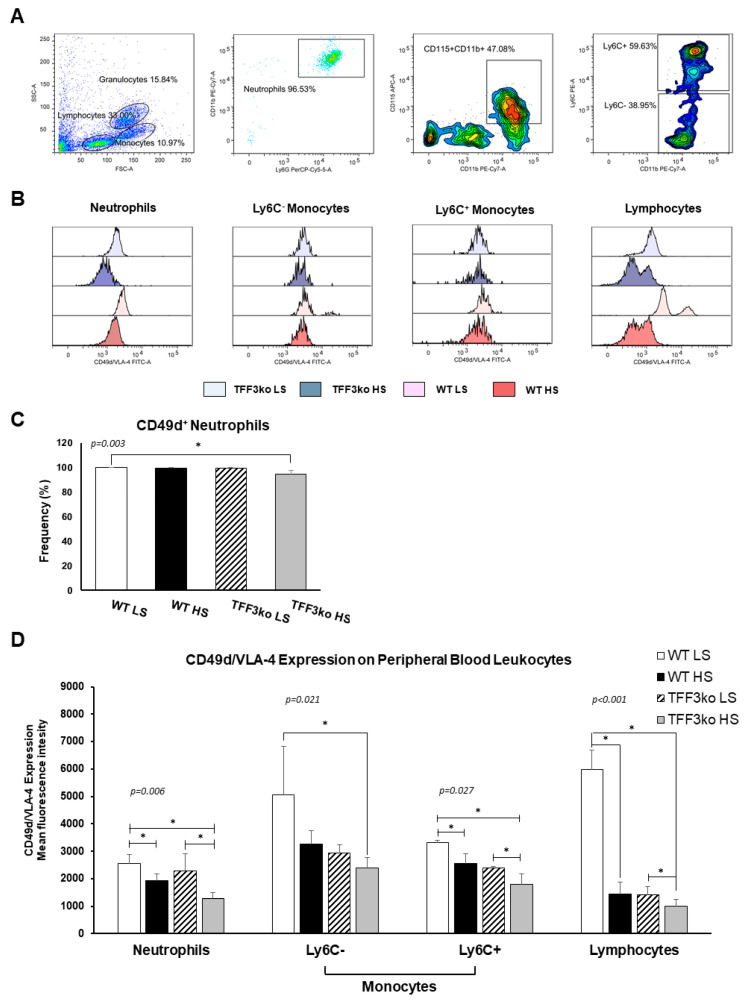
The effect of *Tff3* deficiency and a 7-day high-salt (HS) diet on the integrin very late antigen-4 (VLA-4) in the peripheral blood leukocytes (PBLs). (**A**) demonstrates the representative gating strategy, which included gating on leukocyte subsets based on forward and side scatter. Neutrophils were further defined as Ly6G and CD11b double-positive cells among the granulocyte subpopulation, while monocytes were defined as CD11b^+^CD115^+^ mononuclear cells that were additionally stratified based on the Ly6C expression. (**C**) shows changing rates of CD49d^+^ neutrophils in the study groups following a HS diet. (**B**,**D**) show changing CD49d/VLA-4 expression rates on PBLs in the study groups following a HS diet. HS—high salt; VLA-4—very late antigen-4; PBLs—peripheral blood leukocytes; WT LS—wild-type (C57BL/6N) mice on a low-salt diet; WT LS—wild-type (C57BL/6N) mice on a high-salt diet; TFF3ko LS—*Tff3* knock-out mice on a low-salt diet; TFF3ko HS—*Tff3* knock-out mice on a high-salt diet. Data are presented as mean ± SD and were analyzed by one-way ANOVA or Kruskal–Wallis test where appropriate. In some cases, the effect of the diet on a particular strain (TFFko or WT) was tested by Student’s *t*-test or Mann–Whitney U test; * *p*-values less or equal 0.05 are considered significant.

The gating strategy for CD49d/ very late antigen-4 (VLA-4) expression on peripheral blood leukocytes is shown in Figure 8A. Figure 8B shows histograms representing CD49d/VLA-4 expression on the neutrophils, monocytes, and lymphocytes. The frequency of CD49d/VLA-4 positive neutrophils was similar among the WT LS, WT HS, and TFF3ko LS group, while the HS diet decreased the frequency of CD49d/VLA-4 positive neutrophils in TFF3ko HS compared with the WT LS group only (Figure 8C). In the present study, exposure to the HS diet resulted in a reduced expression of CD49d/VLA-4 in all leukocyte subsets (Figure 8D). TFF3ko mice had lower, albeit not significant, levels of CD49d/VLA-4 expression compared with the WT mice, which were significant only between WT LS and TFFko HS groups in all of the analyzed subsets of leukocytes. However, HS intake significantly suppressed CD49d/VLA-4 expression in all of the subsets of leukocytes compared with their respective controls.

**Figure 9:**
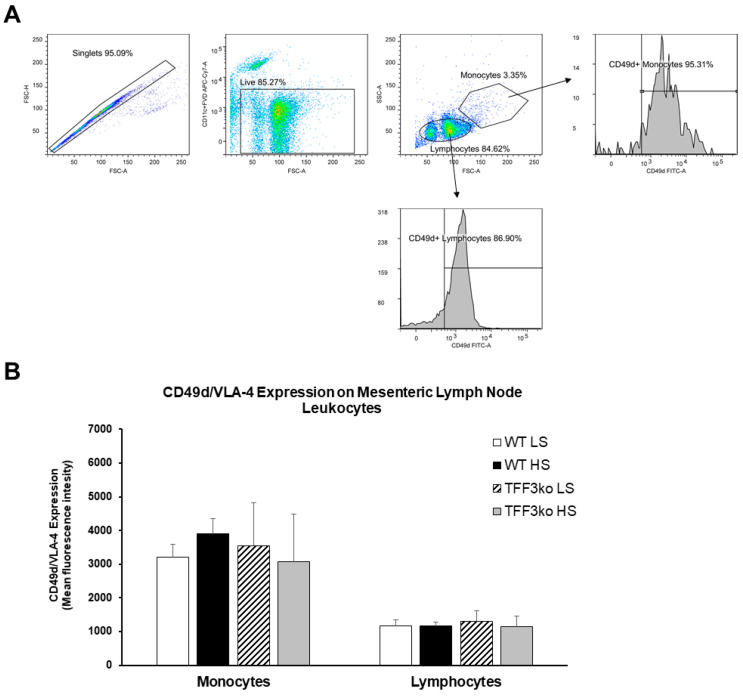
The effect of *Tff3* deficiency and a 7-day high-salt (HS) diet on the integrin very late antigen-4 (VLA-4) on mononuclear cells isolated from mesenteric lymph nodes (MLNs). (**A**) demonstrates the representative gating strategy, which included gating on live single cells followed by gating on lymphocytes and monocytes based on forward and side scatter. (**B**) shows the changing CD49/VLA-4 expression rates in MLNs. HS—high salt; VLA-4—very late antigen-4; MLNs—mesenteric lymph nodes; WT LS—wild-type (C57BL/6N) mice on a low-salt diet; WT LS—wild-type (C57BL/6N) mice on a high-salt diet; TFF3ko LS—*TFff3* knock-out mice on a low-salt diet; TFF3ko HS—*Tff3* knock-out mice on a high-salt diet. Data are presented as mean ± SD and were analyzed by one-way ANOVA or Kruskal–Wallis test where appropriate. In some cases, the effect of the diet on a particular strain (TFFko or WT) was tested by Student’s *t*-test or Mann–Whitney U test. *p*-values less or equal 0.05 are considered significant.

There was no significant difference among the studied groups of mice regarding CD49d/VLA-4 expression on mesenteric lymph node leukocytes (Figure 9A,B).

### 2.4. Genotype and Treatment Interaction Analysis

To differentiate between the genotype × treatment interactions and genotype or treatment effect alone, two-way ANOVA tests were used (Table 1). The serum hsCRP concentration in WT mice increased following the HS diet due to the effects of both the genotype (*p* = 0.05) and the interaction between the genotype and treatment (*p* = 0.031), while changes in IFN-γ, TNF-α, IL-2, and IL-6 showcased that the main influencer was the genotype. A decrease in the rates of CD25 expressing T cells in the MLNs of the TFF3ko group was as a result of both the genotype (*p* = 0.05) and the genotype and treatment interaction (*p* = 0.034). Furthermore, the changes in the rates of γδ-expressing T cells in the peripheral blood in both the knock-out (increased rate) and control mice (decreased rate) was as a result of the genotype and treatment interaction (*p* = 0.001).

Furthermore, regarding the CD11a/LFA-1 expression, it was found that a significant increase in the rates of Ly6C^−^CD11a^high^ monocytes in the peripheral blood was as a result of the interaction between the genotype and treatment (*p* = 0.019), as well as from changes in the Ly6C^−^CD11a^int^ monocytes in MLNs (*p* = 0.033). As for the CD49d/VLA-4 expression, the significant decrease in the peripheral blood CD49^+^ lymphocytes was as a result of the genotype and treatment interaction (*p* = 0.001), while the changes in the other expressing cell subsets were mainly a result of the genotype and/or treatment effect alone, with no significant interaction between the two. A significant effect from the genotype was observed in the rates of CD25 and CD11a/LFA-1 expressing lymphocytes from MLNs (*p* = 0.05 and *p* = 0.05, respectively). Furthermore, the genotype and treatment separately had a significant effect on all CD49d/VLA-4 expressing peripheral blood cells, but not the ones from MLNs.

## 3. Discussion

The salient finding of this research is that changes in the integrin VLA-4 expression in both knock-out and control mice occur mainly as a result of the genotype (TFFko, WT) or treatment (LS, HS) effects alone, while the integrin LFA-1 expression changes only in WT mice due to the effect of interaction between the genotype and the treatment. In addition, the frequency of the γδTCR^+^ expressing T cells significantly differs between the two study groups at the baseline level (i.e., TFFko have a lower cell frequency than WT), while the HS diet has the opposite effect on changing the rates of these CD3^+^ T cells in study groups, by increasing the rates in TFF3ko and decreasing the rates in WT mice. These changes are a result of the genotype and treatment interaction.

The results demonstrate that (1) hsCRP levels increased only in WT mice following salt loading. This suggests that the HS diet promotes an inflammatory response in the control but not in the gene-depleted mice. TFF3 levels are strongly correlated with CRP and other inflammatory markers, so gene depletion suggests a suppressed inflammatory response [37,38]. Furthermore, (2) no changes were detected in any group regarding serum IFN-γ, TNF-α, IL-2, IL-4, or IL-6 concentrations following the HS diet compared to the baseline levels. The IL-6 baseline concentration was significantly higher in the TFF3ko mice compared with the WT group, as well as after salt loading. When looking further into these differences and changes following the HS diet, there seems to be a genetically determined predisposition regarding pro-inflammatory IL-6, as it was higher when lacking *Tff3*. The same effect was observed following a high-fat diet in knock-out mice, where IL-6 upregulation was associated with a sort of a protective role in the absence of *Tff3* when it came to metabolic disorders [39,40,41]. It was also found that (3) CD25 expressing T cell rates (MLN) were significantly higher in TFF3ko mice compared with the baseline rates in WT mice. HS diet and genotype interactions led to a significant reduction in these T cell rates only in the TFF3ko mice. These thymic-derived immunoregulatory cells suppress the progression of the disease through both cytokine-dependent and -independent pathways, making them important for tolerance and prevention [42,43]. (4) γδTCR^+^ expressing CD3^+^ T cells were significantly increased in TFF3ko mice and significantly decreased in WT mice following the HS diet, suggesting a dichotomous response to dietary intervention, which depends on the genotype. Importantly, baseline rates of γδTCR^+^ expressing CD3^+^ T cells in TFF3ko mice were significantly lower compared with their baseline rates in WT mice. γδ T cells promote an inflammatory response, but they also have a role in the activation/accumulation of immunosuppressive cells [44,45]. It seems that these T cell subsets could play an important role in the inflammatory response in *Tff3*-deficient mice. (5) CD11a/LFA-1 expression was significantly increased only in the Ly6C^−^ monocytes of the peripheral blood in WT-HS mice. The expression remained unchanged in the MLNs. On the other hand, (6) CD49/VLA-4 expression was significantly decreased in the peripheral blood cell subsets in both groups following salt loading. Altogether, the results suggest a blunter inflammatory response in *Tff3*-depleted mice following the HS diet, probably due to genotype-determined baseline differences such as higher rates of immunosuppressing CD25 expressing CD4^+^ T cells in MLNs and a changed liver fatty acid (FA) profile.

TFF3 is mainly an exocrine product of the mucous epithelia, although some amounts of TFF peptides are endogenously secreted from lymphoid tissues and organs [35,46]. It has been suggested that anti-inflammatory cytokines such as IL-4 and IL-13 upregulate *Tff3* expression through effects on the signal transducer and activator of transcription 6 (STAT6) transcription factor, while pro-inflammatory TNF-α, IL-6, and IL-1β inhibit its transcription (via NF-κB pathway) and downregulate the *Tff3* expression [35,47,48,49]. Furthermore, it has been suggested that treatment with recombinant human *Tff3* significantly decreases the activity of NF-κB, as well as alleviates inflammation [50]. Earlier studies have demonstrated that *Tff3* deficiency in mice causes (a) better glucose utilization; (b) an increased number of lipid droplets containing vesicles, which serve as reservoirs of fatty acids, phospholipids and sterols in hepatocytes and, consequently; and (c) a change in the FA profile/ratios in the liver [32,51]. These changes in lipid metabolism are likely to underlie the increased antioxidant activity, better glucose tolerance, and vascular response after the HS diet in knock-out mice compared with the control [31].

It is well documented that the HS diet promotes hypertension and arterial fibrosis and amplifies inflammatory response, further causing severe vascular impairments in Sprague-Dawley (SD) rats following salt loading [52]. HS intake resulted in increased protein expression levels of pro-inflammatory VEGF, IL-1β, IL-6, and TNF-α, and upregulation of STAT3 transcription factor [35]. Furthermore, similar results were obtained in Dahl Salt-Sensitive (SS) rats during a 5-week HS intake, which led to hypertension, as well as increased mRNA levels of TNF-α, IL-6, and IL-1β [53]. Increased levels of pro-inflammatory cytokines were previously reported in hypertensive humans and hypertensive rat models (e.g., angiotensin II-induced hypertensive rat and spontaneously hypertensive rat, SHR) [54,55,56,57]. Similarly, HS intake in mice resulted in an enhanced inflammatory response in terms of cytokine production (pro-inflammatory TNF-α, IL-17A, and IL-23), increased IL-23R^+^CD4 T cells, MAP, and exacerbated colitis in mice with artificially induced inflammatory bowel disease (IBD) [4,58]. Several studies in Dahl SS and SHRs reported cytokine secretion and an overexpression of leukocyte adhesion molecules, including ICAM-1, MCP-1, and Mac-1 in events of hypertension and endothelial dysfunction development following salt intake [59,60,61]. In our study, although the HS diet did not exert a significant effect on the serum concentrations of IFN-γ, TNF-α, IL-2, IL-4, and IL-6, we observed that the changes that happened in serum concentrations of IFN-γ, TNF-α, and IL-2 were solely a result of the genotype. In both study groups, we observed a slight decrease in IL-2 and an increase in TNF-α concentrations following the HS diet, while the IFN-γ serum concentrations increased in the knock-out and decreased in the control mice.

Interestingly, Yilmaz et al. (2012) found that in primary hypertension patients, systolic (SBP) and diastolic blood pressure (DBP) was not changed significantly with salt intake, while CRP and urinary albumin levels were significantly higher in the HS intake group, suggesting enhanced inflammation in those individuals [62]. Our results are in agreement with this study since hsCRP serum concentrations were significantly increased in WT mice after salt-loading, while, although not significant, the concentration decreased in TFFko mice. Further analysis showed that these changes resulted from genotype and treatment interaction, suggesting protective anti-inflammatory conditions in TFFko mice.

LFA-1 and VLA-4 represent integrin receptors, with the first being expressed solely on mature leukocytes, and the latter being expressed on early hematopoietic stem progenitor cells, lymphocytes, monocytes and eosinophils [63,64,65]. They participate in leukocyte trafficking and are involved in the leukocyte-endothelial cell adhesion cascade, while also contributing to cell-cell interactions [65,66]. LFA-1 and VLA-4 paired with their respective ligands on endothelial cells, ICAM-1 and VCAM-1, play a pivotal role in endothelial function through effects on activation of leukocytes, progression of leukocyte migration and release of cytokines, navigating the inflammatory response in the direction of the increase or mitigation of inflammation [67,68]. Mihalj et al. [9] showed that salt-loading altered peripheral blood leukocyte phenotype and dynamics in both humans and Sprague-Dawley (SD) rats. In addition, HS intake decreased the expression of LFA-1 and VLA-4 in healthy human subjects. Furthermore, the same dietary protocol reduced VLA-4 expression but increased LFA-1 expression in rats. This is in line with the present results, as salt loading in the control mice (WT) resulted in an increased CD11a/LFA-1 expression in the peripheral blood monocytes, while no changes were detected in knock-out mice in the HS diet, and that effect was as a result of the interaction between the genotype and treatment. Similarly, Dahl SS rats fed a high-salt diet had increased leukocyte adhesion due to an increase in MCP-1 and ICAM-1-related adhesion molecules in the kidney [59]. Previously investigated by our research group, short-term NaCl dietary intake caused an increased frequency of activated CD11b-expressing cells and an increased expression of total CD11b/c (in granulocytes and CD3 lymphocytes) in both SD rats and humans [5].

In addition, in the present study, the HS diet decreased the CD49d/VLA-4 expression in the peripheral blood neutrophils, monocytes, and lymphocytes in both the control and *Tff3* knock-out mice as a result of the genotype and treatment effects. Peripheral blood (circulating) leukocyte integrins were kept in an inactive form until they reached the spot of inflammation, where adhesion molecule expression was induced after cell activation by IL-1 and TNF-α [69].

The HS diet in our study decreased CD25^+^CD4^+^ lymphocyte rates in the MLNs of *Tff3* knock-out mice, with no changes detected in the control group. A decrease in these particular rates in our animals could, however, be explained by the effects of the changed fatty acid metabolism. Some fatty acids (e.g., docosahexaenoic acid (DHA)) have an inhibitory effect on regulatory T cells [70]. As mentioned above, the TFF3ko mice had a modified FA metabolism, and most importantly, exerted increased levels of PUFAs in the liver, namely eicosadienoic acid, AA, and DHA [32]. Their liver n-3/n-6 PUFAs ratio was increased while the sera ratio was reduced. Interestingly, the HS diet activated Th17 cells through effects on IL-17A and IL-23 [71]. Thus, in light of these results, one can speculate that the HS diet probably changed the frequencies of Th17 cells and potentially increased the rates of IL-17A-expressing T cells at the expense of CD4^+^CD25^+^ T cells in the knock-out mice [43,72,73]. As the knock-out mice had significantly higher frequencies of CD25-expressing T cells than the control mice, the effect was more drastic following the HS diet.

*Tff3* regulates innate immune response at the mucosal surfaces. Furthermore, γδT cells (1–10% in peripheral blood) play an important role in mucosal leukocyte response, serving as a link between innate and adaptive immune systems, providing regulation through IL-17 and IFN-γ secretion [74,75]. Their rapid immune response to stimuli includes the generation of large amounts of pro-inflammatory IL-17, IL-21, and INF-γ, further amplifying the generation of Th17 cells, as well as their own generation [75,76]. In the present study, the rates of peripheral blood CD3^+^γδTCR^+^ T cells were significantly increased following salt intake only in TFF3ko mice. It was shown that these changes resulted from the genotype and treatment interactions. A pilot study on healthy male participants stated that the HS diet had no profound effect on γδTCR T cells after 2 weeks, although there were some changes observed regarding early activation markers, such as increased CD69-expression in γδ1 T cells [77]. Even though not significant, we observed the genotype-conditioned increase of INF-γ serum concentration in knock-out mice following the HS diet. This may be the path to focus on when investigating *Tff3* knock-out mice immune responses, as both TFF3 and γδ T cells are closely related to the mucosal immune response.

## 4. Materials and Methods

### 4.1. Dietary Protocols

**Figure 10:**
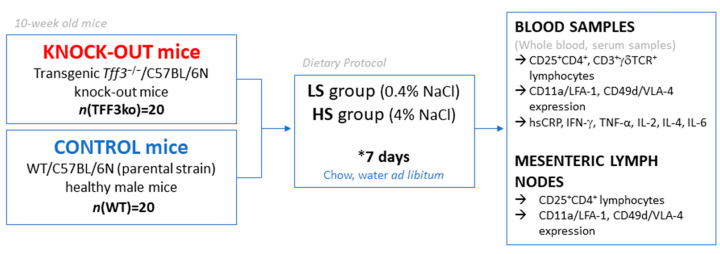
Schematic representation of the study design.

Schematic presentation of the study design is shown in Figure 10. The animals were housed in standard plastic cages, in a temperature and humidity-controlled environment, with a 12:12 h light–dark cycle and they had access to water and chow *ad libitum* at the animal care facility (nationally registered and certified user/breeder of mice and rats for educational and scientific purposes) of the Faculty of Medicine at the Josip Juraj Strossmayer University of Osijek, Croatia. Transgenic *Tff3*^−/−^/C57BL/6N knock-out (TFF3ko) mice (*n* = 20) and wild-type WT/C57BL/6N (WT) (parental strain) healthy male mice (*n* = 20) were divided evenly into two groups: low-salt (LS) and high-salt (HS) groups. The LS group was fed standard rodent chow (0.4% NaCl), while the HS group was fed food containing 4% NaCl for 7 days from 10 weeks of age. The housing conditions, animal welfare, and protocols were reviewed and approved by the Institutional as well as the National Local Ethical Committee. The origin of the mice used in this study was previously described by Bujak et al. (2018) [32].

### 4.2. Sampling and Isolation

Blood samples were collected in tubes without an anticoagulant so as to obtain serum and whole blood samples. Blood was centrifuged at 3500 rpm for 10 min to separate the serum from the blood cells. Samples were stored at −80 °C until they were used. Whole blood samples were used for immunophenotyping purposes. Mesenteric lymph nodes (MLNs) were extracted and macerated using two slides, washed in 1× PBS, and passed through cotton wool into a sterile tube. After two-step rinsing and centrifugation, the samples were prepared for further use. All of the measurements were performed in the Laboratory for Vascular Physiology and the Laboratory for Molecular and Clinical Immunology, at the Department of Physiology and Immunology, Faculty of Medicine Osijek, Josip Juraj Strossmayer University of Osijek, Osijek (Croatia).

### 4.3. Assay of Protein Concentration in Serum

The serum concentration of the high-sensitivity CRP, IFN-γ, TNF-α, IL-2, IL-4, and IL-6 were measured with antibody-based, magnetic bead reagent kits (Invitrogen ProcartaPlex kits; Invitrogen by Thermo Fisher Scientific, Waltham, MA, USA) and panels for multiplex protein quantitation using the Luminex 200 instrument platform (Luminex Corp., Austin, TX, USA), according to the manufacturer’s instructions. Measurements were performed at the Laboratory of Molecular and HLA Diagnostics Osijek University Hospital, Osijek, Croatia. Quantitation was done in ProcartaPlex Analyst v1.0 free software (eBioscience, Affymetrix by Thermo Fisher Scientific, Waltham, MA, USA) and expressed as concentration in picograms per millilitre.

### 4.4. Flow Cytometry

Flow cytometry measurements were performed after 7 days of the dietary protocol with an appropriate antibody mixture depending on the cell subset or expression of interest. The rates of CD25^+^CD4^+^ lymphocytes were measured in the peripheral blood and mesenteric lymph nodes, while the rates of CD3^+^γδTCR^+^ lymphocytes were measured only in the peripheral blood. The expressions of CD11a/LFA-1 and CD49d/VLA-4 were measured in the peripheral blood leukocyte subsets (neutrophils, monocytes, and lymphocytes). Furthermore, the expression of LFA-1/CD11a was also measured in the lymphocytes and monocytes from the mesenteric lymph nodes. Measurements of stained samples were carried out using a BD FACS Canto II cytometer (FACSCanto II, Becton Dickinson, San Jose, CA, USA) equipped with blue Argon 488 nm and Red HeNe 633 nm laser lines. Data analysis and visualization were performed using the FlowLogic software (Inivai Technologies, Mentone, Australia).

### 4.5. Statistical Analysis

The differences among groups were assessed using the one- and two-way ANOVA tests (GraphPad Prism; Microsoft Excel 2016). Student’s *t* test was used to test the differences in normally distributed numerical variables between the two groups, while in the case of deviations from the normal distribution, the Mann–Whitney U test was used (SigmaPlot version 11.2, Systat Software, Inc., Chicago, IL, USA). The results are presented as mean ± SD in the tables and in the graphs as arithmetic mean ± SD. The level of significance was determined at *p* < 0.05.

## 5. Conclusions

The results of the present study partially confirmed earlier speculations that a high-salt dietary intake has a milder effect on mice with *Tff3* gene depletion compared with wild-type mice. It has also opened the door to new research venues, particularly regarding the fatty acid profile and its role in the alleviation of the inflammatory response. Further investigation should also focus on the interplay between γδ T cells and *Tff3*, as well as their effects on the mucosal immune response following dietary salt intake.

## Figures and Tables

**Table 1:** Genotype and treatment (HS diet) interaction effects on cell adhesion molecule expression (two-way ANOVA).

Expression	Rates (%) of Expressing Cells	Group (Genotype; Treatment)				Significance Level (*p* < 0.05)		
		WT LS	WT HS	TFF3ko LS	TFF3ko HS	Genotype	Treatment	Genotype × Treatment
*CD25*	CD4^+^CD25^+^ (MLN)	7.94 ± 1.1	9.44 ± 0.9	13.7 ± 1.9	8.22 ± 3.4	0.05 *	0.274	0.034 *
	CD4^+^CD25^+^	5.71 ± 0.9	4.89 ± 1.7	5.11 ± 1.8	4.89 ± 2.45	0.794	0.577	0.362
*γδ*	CD3^+^γδTCR^+^	14.92 ± 4.8	4.9 ± 2	3.54 ± 2.8	13.89 ± 1.9	0.636	0.942	0.001 *
*CD11a/LFA-1*	CD11a^+^ neutrophils	1665.3 ± 415.4	2115.9 ± 257.3	1962.41 ± 252.9	2146.67 ± 260.6	0.229	0.152	0.297
	Ly6C^+^CD11a^+^ monocytes	1614.58 ± 220.3	1689.23 ± 202.6	1618.23 ± 318.1	1970.6 ± 293.4	0.376	0.197	0.387
	Ly6C^−^CD11a^high^ monocytes	5374.66 ± 91.2	5959.37 ± 258.5	6145.22 ± 246.4	5473.32 ± 685.5	0.529	0.846	0.019 *
	Ly6C^−^CD11a^int^ monocytes	1282.39 ± 29.9	1342.29 ± 66.4	1359.35 ± 37.7	1309.31 ± 138.3	0.648	0.918	0.270
	CD11a^high^ lymphocytes	2682.85 ± 183.1	2815.28 ± 228.9	2714.47 ± 85.9	2814.10 ± 197.7	0.888	0.301	0.879
	CD11a^int^ lymphocytes	581.20 ± 134.9	676.32 ± 210.1	578.63 ± 51.7	540.18 ± 81.7	0.396	0.723	0.413
	CD11a^int^ monocytes (MLN)	1605.43 ± 18.7	1777.77± 148.4	1733.24 ± 31.3	1693.33 ± 94	0.909	0.379	0.033 *
	CD11a^high^ monocytes (MLN)	5269.45 ± 46.1	5389.06 ± 261.8	5502.09 ± 129.9	5407.61 ± 313.6	0.176	0.857	0.472
	CD11a^+^ lymphocytes (MLN)	1246.07 ± 43.4	1295.63 ± 93.1	1143.84 ± 122.9	1132.88 ± 116.4	0.05 *	0.787	0.645
*CD49d/VLA-4*	CD49d^+^ neutrophils	2459.03 ± 320.2	1724.74 ± 193.4	2125.02 ± 539.5	1056.57 ± 125.5	0.032 *	0.002 *	0.412
	Ly6C^−^CD49^+^ monocytes	3919.15 ± 942.8	3115.04 ± 483.9	2821.59 ±301.1	2135.75 ± 304.7	0.014 *	0.05 *	0.862
	Ly6C^+^CD49^+^ monocytes	3045.18 ± 122.2	2334.93 ± 397.7	2215.57 ±80.9	1390.12 ± 57.3	<0.0001 *	0.0003 *	0.653
	CD49^+^ lymphocytes	4136.31 ± 675.2	1305.85 ± 418.3	1182.81 ± 381.8	783.42 ± 93.1	0.0001 *	0.0002 *	0.001 *
	CD49^+^ monocytes (MLN)	2065.4 ± 227.3	2497.41 ± 278.6	2348.79 ± 878.9	2034.01 ± 841.2	0.803	0.878	0.273
	CD49^+^ lymphocytes (MLN)	1048.74 ± 161.6	1038.7 ± 104.3	1176.37 ± 266.9	1024.57 ± 279.9	0.609	0.468	0.524
*Serum concentrations*	C-reactive protein	1079.75 ± 464.2	2305.84 ± 1052.4	1439.54 ± 390.9	1117.75 ± 342.3	0.074	0.05 *	0.031 *
	Interferon gamma	124.5 ± 87.5	108.2 ± 65.9	150.41 ± 108.5	220.70 ± 122.8	0.032 *	0.389	0.175
	Tumor necrosis factor alpha	129.15 ± 75.5	179.59 ± 66.8	189.88 ± 111.8	241.53 ± 128.9	0.05 *	0.112	0.985
	Interleukin 2	112.55 ± 29.8	94.65 ± 36.4	157.29 ± 87.3	134.81 ± 53.3	0.022 *	0.264	0.898
	Interleukin 4	65.98 ± 46.1	94.17 ± 40.3	73.15 ± 51.4	63.95 ± 25.9	0.391	0.479	0.168
	Interleukin 6	93.38 ±26.4	109.61 ± 22.3	150.03 ± 35.3	129.70 ± 28.3	0.0003 *	0.616	0.088

Data presented as mean ± SD are compared by two-way ANOVA, followed by Bonferroni post hoc test; * *p* ≤ 0.05 is considered statistically significant (*p* values for the effect of genotype, treatment, or genotype and treatment interaction are presented separately). PBLs—peripheral blood leukocytes; MLNs—mesenteric lymph nodes; WT—wild type; TFF3ko—*Tff3* knock out; LS—low salt; HS—high salt.

## Data Availability

Data is contained within the article.
